# The role of AMPK in cancer metabolism and its impact on the immunomodulation of the tumor microenvironment

**DOI:** 10.3389/fimmu.2023.1114582

**Published:** 2023-02-15

**Authors:** Chenicheri Kizhakkeveettil Keerthana, Tennyson Prakash Rayginia, Sadiq Chembothumparambil Shifana, Nikhil Ponnoor Anto, Kalishwaralal Kalimuthu, Noah Isakov, Ruby John Anto

**Affiliations:** ^1^ Division of Cancer Research, Rajiv Gandhi Centre for Biotechnology, Thiruvananthapuram, Kerala, India; ^2^ Department of Biotechnology, University of Kerala, Thiruvananthapuram, Kerala, India; ^3^ The Shraga Segal Department of Microbiology, Immunology and Genetics, Faculty of Health Sciences, Ben-Gurion University of the Negev, Beer Sheva, Israel

**Keywords:** AMPK signaling, cancer metabolism, tumor microenvironment, immune response, T cells, macrophages, immune checkpoints, phytochemicals

## Abstract

Adenosine monophosphate-activated protein kinase (AMPK) is a key metabolic sensor that is pivotal for the maintenance of cellular energy homeostasis. AMPK contributes to diverse metabolic and physiological effects besides its fundamental role in glucose and lipid metabolism. Aberrancy in AMPK signaling is one of the determining factors which lead to the development of chronic diseases such as obesity, inflammation, diabetes, and cancer. The activation of AMPK and its downstream signaling cascades orchestrate dynamic changes in the tumor cellular bioenergetics. It is well documented that AMPK possesses a suppressor role in the context of tumor development and progression by modulating the inflammatory and metabolic pathways. In addition, AMPK plays a central role in potentiating the phenotypic and functional reprogramming of various classes of immune cells which reside in the tumor microenvironment (TME). Furthermore, AMPK-mediated inflammatory responses facilitate the recruitment of certain types of immune cells to the TME, which impedes the development, progression, and metastasis of cancer. Thus, AMPK appears to play an important role in the regulation of anti-tumor immune response by regulating the metabolic plasticity of various immune cells. AMPK effectuates the metabolic modulation of anti-tumor immunity *via* nutrient regulation in the TME and by virtue of its molecular crosstalk with major immune checkpoints. Several studies including that from our lab emphasize on the role of AMPK in regulating the anticancer effects of several phytochemicals, which are potential anticancer drug candidates. The scope of this review encompasses the significance of the AMPK signaling in cancer metabolism and its influence on the key drivers of immune responses within the TME, with a special emphasis on the potential use of phytochemicals to target AMPK and combat cancer by modulating the tumor metabolism.

## Introduction

Cancer metabolism relies on the fundamental principle of metabolic reprogramming which leads to the malignant transformation of normal cells into cancer cells. The acquisition of these metabolic alterations is regarded as one of the hallmarks of cancer. Metabolic reprogramming not only affects the biological activity of tumor cells, but it also regulates the differentiation and function of various immune cell populations. Previous literature suggests that the metabolic status of immune cells is crucial for the functional plasticity of the immune cells (1, 2). Over the past few decades, there has been a growing appreciation of the complex molecular crosstalk between the components of the TME and the cellular metabolic pathways. Besides, there is a growing body of evidence suggesting the chemoresistance and malignant progression of tumors to be a manifestation of the metabolic reprogramming that occurs in the immune cells present within the tumor microenvironment (2).

## AMPK: An overview

Adenosine monophosphate (AMP)-activated protein kinase (AMPK), is an evolutionarily conserved serine/threonine kinase and the primary regulator of cellular energy homeostasis. AMPK functions as a key metabolic sensor and is actively involved in all planes of energy metabolism and mitochondrial biogenesis (3). AMPK is activated in response to depletion in cellular energy levels resulting from conditions, such as hypoxia and nutrient starvation (4). Upon activation, it promotes ATP production and thus maintains cellular energy homeostasis.

**Figure 1:**
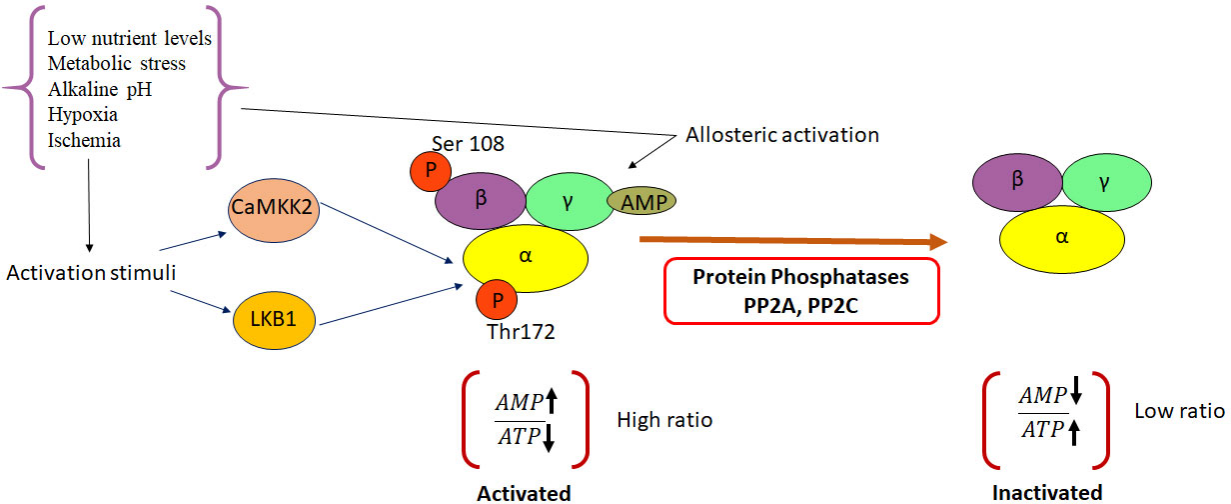
Molecular mechanisms leading to activation and inactivation of AMPK. AMPK is phosphorylated by the two upstream kinases, LKB1 and CAMKKβ in response to various activation stimuli. Protein phosphatases PP2A and C cause de-phosphorylation of AMPK and render it inactive. CAMKK2, Ca^2+^/Calmodulin-dependent protein kinase kinase 2; LKB1, Liver kinase B1; AMP, Adenosine mono phosphate; PP2A, protein phosphatase 2; PP2C, protein phosphatase 2C; ATP-Adenosine triphosphate.

The metabolic stress induced in cells as a result of increase in the cellular AMP : ATP ratio, due to decreased ATP production, and high levels of intracellular AMP, consequently, leads to the activation of AMPK. The resulting allosteric changes promote the phosphorylation of threonine 172(Thr^172^) within the activation loop of the AMPK catalytic α-subunit (5). This occurs following the binding of AMP/ADP to the γ-regulatory subunit of AMPK. The phosphorylation is mediated by upstream kinases, including liver kinase B1 (LKB1) and calcium/calmodulin-dependent protein kinase kinase (CaMKKβ). Although allosteric activation is triggered only by the binding of AMP, the other two complementary effects can be mimicked by ADP (6). AMPK can sense even subtle changes in AMP concentrations (7–9). The binding of AMP to AMPK inhibits the dephosphorylation of the α-subunit of AMPK and helps maintaining AMPK in its activated state (10). The activation of AMPK in response to energy stress, further, leads to the concomitant inhibition of anabolic pathways and promotion of catabolic pathways that are down-stream of AMPK (11). However, under energy-replete conditions, enzymes such as TGFβ-activated kinase 1 (TAK1); protein phosphatase 2A (PP2A); protein phosphatase 2C (PP2C) and Mg^2+^/Mn^2+^-dependent protein phosphatase 1E (PPM1E) render AMPK inactive by maintaining it in an unphosphorylated state (12). A schematic representation highlighting the mechanism of AMPK activation and inactivation is depicted in **Figure 1**.

Given the central role of AMPK in metabolism and the plethora of down-stream metabolic processes that are regulated by this kinase, AMPK has attracted global attention as a potential target for treating metabolic diseases such as obesity, Type 2 diabetes and cancer. Literature review suggests the de-regulation of cellular metabolism to be one of the key drivers of tumorigenesis and cancer progression (13). Besides, recent reports state that AMPK plays a central role in tumor cellular bioenergetics and in evoking an anti-tumor immune response, owing to its molecular crosstalk with various key players of the tumor microenvironment (TME). This review focuses on the role of AMPK in cancer metabolism and AMPK-mediated immunomodulation of the TME, highlighting AMPK as a therapeutic target in cancer patients. Furthermore, we discuss the use of plant-derived anti-cancer agents as activators of AMPK to combat cancer by modulating the tumor metabolism.

## Structure of AMPK

Structurally, the protein kinase AMPK is a heterotrimeric αβγ complex, which comprises of a catalytic α-subunit in association with β and γ regulatory subunits (11, 14). The complex structure of the γ -subunit, along with the non-catalytic C-terminal fragments of α and β subunits, in *S. pombe* was first reported by Townley (3).The β-subunit consists of a glycogen-binding domain (GBD), while the γ-subunit contains Bateman domains responsible for nucleotide binding. AMPK γ-subunit contains four tandem repeats of sequence motifs known as cystathionine-b-synthase (CBS). In AMPK subunits, these repeats assemble to a disc-like shape with one repeat in each quadrant, generating four nucleotide-binding sites. Of these, AMP, ADP, and ATP bind in competition at sites 1 and 3, while site 2 remains vacant. AMP is permanently bound to site 4 (7, 15).

Even though all eukaryotes have homologs of these proteins, the number of subunit genes vary between organisms (11). The aspartate residue (Asp^139^) in the catalytic loop functions as the base for catalysis, while mutation of aspartate to alanine renders AMPK catalytically inactive. AMPK belongs to a class of protein kinases that are basophilic and hence require the presence of basic residues in the N-terminal sequence, adjacent to the site of phosphorylation on target proteins (16).

Following the sequencing of the mammalian AMPK, it was found to be a homolog of the *Saccharomyces cerevisiae* protein kinase SNF1, which was previously identified as a regulator of gene transcription in response to glucose starvation (3, 16–19). The three alternative subunits of AMPK can form up to 12 distinct isoforms (14). There are two α subunit isoforms in mammalian AMPK and one in *S.cervisea*, which has an N-terminal kinase domain followed by an autoinhibitory region, plus a C-terminal domain involved in complex formation. β-subunits have two isoforms in mammalian AMPK and three in *S.cervisea*. Those subunits contain a canonical Ser/Thr kinase domain (KD), an adenine nucleotide sensor segment, termed as linker, an autoinhibitory domain (AID) and a subunit-interacting C-terminal domain (CTD), the latter of which contains the serine/threonine rich activation loop containing phosphorylation sites for various downstream proteins (11, 20–22). There are three γ-subunit isoforms in mammalian AMPK and one in *S.cervisea*. The γ- subunits have four copies of CBS motifs preceding unrelated N-terminal extensions of varying lengths. The CBS motifs function in pairs to form a discrete structural unit called a Bateman domain which are the regulatory binding sites for AMP and ATP (11, 16).

**Figure 2:**
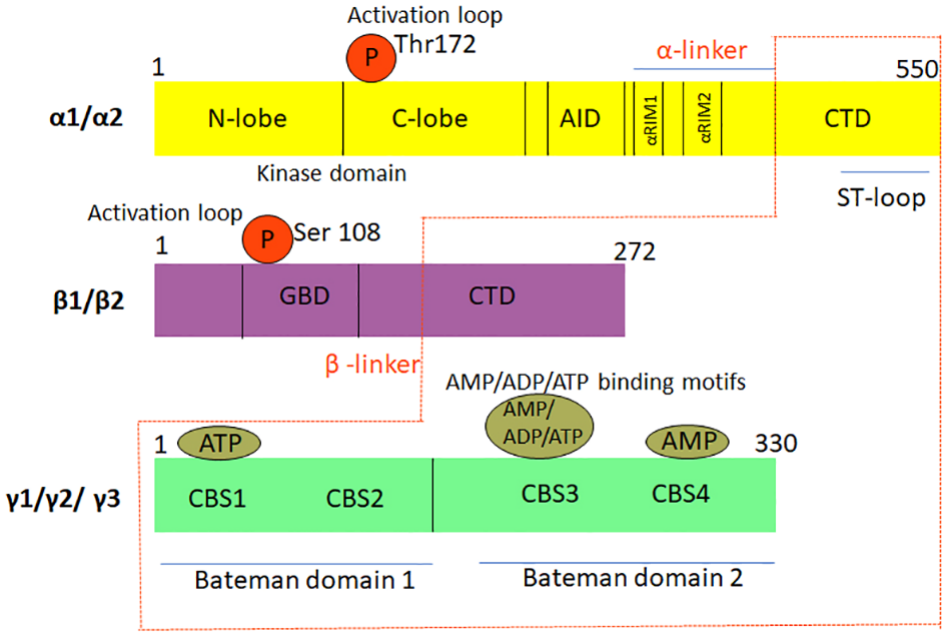
Graphical representation of the domain structure and isoforms of AMPK. The three sub-units, namely, αβγ, which constitute the heterotrimeric complex structure of AMPK have different isoforms. While the α and β sub-units have two isoforms each, the γ sub-unit has three isoforms. Thr, threonine; Ser, Serine; AID, Autoinhibitory domain; ST-loop, Stem loop; CTD, C-terminal domain; αRIM1/2, α-subunit motifs; α/β linker, regulatory linker region; GBD, GTPase protein binding domain; CBS1 domain, Cystathionine beta synthase1 domain; CBS2 domain, Cystathionine beta synthase2 domain; CBS3 domain, Cystathionine beta synthase3 domain; CBS4 domain, Cystathionine beta synthase 4 domain AMP, Adenosine monophosphate; ADP, Adenosine diphosphate; ATP, Adenosine triphosphate.

The structural and functional modules of the three AMPK subunits share a high degree of amino acid similarity. The α1- and α2-subunits share a sequence identity of 77%, which increases to 90% for the protein kinase module and 61% in the C-terminus. The α-subunits share 60% sequence identity with the kinase domain of sucrose non-fermenting 1 (SNF1) and 46% identity with the entire subunit (16). The CBM motifs of β1 and β2 retain ~80% identity, while the CBS modules across the three γ isoforms share ~60% conservation. Some of the AMPK subunits are known to be alternately spliced in different tissues allowing for further regulation and complexity (5, 16). This basic architecture is conserved in the *S. cerevisiae* as well as in mammalian homologs (3). **Figure 2** is a graphical representation of the domain structure and the isoforms of AMPK.

## Physiological roles of AMPK

AMPK activity governs a plethora of metabolic and physiological processes. This kinase is dysregulated in various chronic diseases, such as diabetes, neurodegenerative and neuromuscular diseases, cardiovascular diseases, and cancer (23). The following section substantiates some of the key findings on the physiological roles of AMPK under normal and disease conditions:

### Metabolic homeostasis

Being a multi-substrate serine/threonine kinase, AMPK provides binding sites for an array of regulatory nucleotides. The primary physiological manifestation of activated AMPK is to redirect metabolism towards increased catabolism and decreased anabolism through the phosphorylation of key proteins in response to metabolic stress. This metabolic reprogramming is initiated in order to ensure the replenishment of intracellular ATP levels back to normal (12, 13).Studies using AMPKα1 and α2 knockout mouse models by Violett et al., revealed that glucose homoeostasis was unaltered in AMPKα1^−/−^ mice whereas, high plasma glucose levels and low plasma insulin concentrations were observed in AMPKα2^−/−^ mice although the insulin secretion was not altered in both types of mice. Furthermore, the team also reported that the AMPKα2 catalytic subunit modulates the activity of the autonomous nervous system *in vivo (* 24).

### Type 2 diabetes mellitus

Accumulation of glucose, fatty acids, and amino acids have been reported to suppress AMPK and eventually lead to insulin resistance, while stimulation of AMPK activity improved blood glucose levels, suggesting that development of AMPK activators might potentially function as anti-diabetic drugs (25–27). Metabolic inflexibility and insulin resistance present in Type 2 diabetes mellitus and obesity develop as a result of dysfunctional mitochondria in the muscles (28, 29).

### Neurodegenerative and Neuromuscular diseases

Activation of AMPK in skeletal muscles induces peroxisome proliferator-activated receptor γ coactivator 1α (PGC1α) and up-regulates mitochondrial genes (30). These observations suggest that AMPK activators might have a beneficial effect in combating metabolic disorders. Studies also reveal a negative correlation between inflammation and AMPK. The proinflammatory cytokine, tumor necrosis factor α (TNFα), suppresses the phosphorylation of AMPK and increases the expression of protein phosphatase 2C (PP2C) in the skeletal muscles thereby inducing insulin resistance (31).

Interestingly, AMPK has been reported to play a key role in the development of Alzheimer’s disease (AD), a progressive neurodegenerative disease. One of the hallmark features of AD includes the aberrations in β-amyloid metabolism. Previous studies have revealed that AMPK activation is closely linked with the aberrant processing of β-amyloid protein precursor (AβPP). Further, AMPK signaling controls β-amyloid metabolism *via* the suppression of Glycogen synthase kinase 3β (GSK-3β) (32). Another independent study indicates that AMPK hyperphosphorylation occurs in the brains of mice that have experimental AD, as well as human patients suffering from AD (33, 34). Further studies demonstrated that resveratrol lowers extracellular accumulation of β-amyloid peptides through the activation of AMPK. This study also demonstrated that the inhibition of mammalian target of rapamycin complex 1 (mTORC1) mediated by the activation of AMPK induces autophagy and lysosomal degradation of the β-amyloid peptide (35).

### Cardiovascular diseases

AMPK is also activated under conditions of cardiac ischemia and studies indicate that the intrinsic activation of AMPK protects the heart from injuries induced due to the ischemia (36, 37). Treatment with AMPK activators such as 5-Aminoimidazole-4-carboxamide ribonucleotide (AICAR) and metformin brought about a significant decrease in contractile dysfunction, apoptosis, and fibrosis in canine heart failure models (38).

### Cancer

AMPK activation has been extensively investigated as a potential therapeutic target in combating different types of cancer. AMPK phenotypically functions as a tumor suppressor by resisting pro-tumorigenic metabolic processes and directly inducing cell-cycle arrest in cancer cells. Cyclooxygenase 2 (COX-2) plays an important role in cancer stemness. Interestingly, AMPK also acts as a COX-2 inhibitor in cancers of the breast and colon (39). AMPK activation is critical in alleviating metabolic and energetic stresses associated with tumor progression. Besides, AMPK activation triggers the onset of multiple cell death mechanisms. It influences the cell cycle checkpoints, autophagy, mitophagy, and apoptosis. AMPK promotes autophagy and mitophagy by activating UNC-51-like kinase 1 (ULK1) and death-associated protein 1 (DAP1) respectively (40, 41). AMPK initiates the apoptotic program *via* the activation of p53, p21, and p27. It manifests cell cycle arrest *via* the inhibition of HUR and the concomitant activation of Cyclins A, B1, and D1 (42). Previous reports indicate that metformin, an AMPK activator down-regulates c-MYC in an AMPK-dependent manner in breast cancer cell lines (43).There are several other reports highlighting the significance of AMPK in cancer chemoprevention (44–50). Studies using MT 63–78, a specific and potent direct AMPK activator have revealed that AMPK activation induces mitotic arrest and apoptosis in androgen-sensitive and castration-resistant prostate cancer *via* mTORC1 blockade and the suppression of *de novo* lipogenesis (51). Previous studies have documented that treatment of hepatocellular carcinoma cells with the AMPK activators, AICAR and metformin, significantly inhibited their proliferation, and induced cell cycle arrest at the G1-S phase (52). Zou et al. demonstrated that metformin-induced activation of AMPK down-regulated the expression of segment polarity protein dishevelled homolog3 (DVL3), a key oncoprotein that activates the Wnt/β-catenin signaling pathway (53). Down-regulation of DVL3 reduced the levels of β-catenin and its downstream targets, cyclin D1 and c-Myc, resulting in suppression of cell proliferation. AMPK activation has also been reported to induce p53-dependent apoptotic effects in breast cancer cells (9),and inhibit the metastatic potential of melanoma cells by modulating the ERK signaling pathway and reducing the levels of the COX-2 protein (54). It also induces autophagic and apoptotic cell death through AMPK/JNK signaling (55).Vara-Ciruelos et al. have demonstrated the tumor suppressor role of AMPK-α1 by utilizing T-cell-specific knock-outs of *Pten* and *Prkaa1* gene encoding AMPK-α1. In these models, absence of *Pten* and *Prkaa1*genes promoted lymphoma development at an early age and the tumors were significantly more aggressive (56).Previous literature also highlights the impacts of AMPK activation in augmenting the chemosensitizing efficacy of natural and synthetic compounds in combination with conventional chemotherapeutics (57–64). A list of various pharmacological activators of AMPK and their implication in different types of cancer is included in **Table 1**.

**Table 1:** Pre-clinical evidences of the anti-cancer effects of pharmacological activation of AMPK.

Name of the compound	Type of cancer	Remarks	Reference
Metformin	Colorectal	AMPK activation suppresses cancer stem cells by preventing prenylation of the mevalonate pathway proteins AMPK activation and elevated levels of ROS together suppress the mTOR pathway and its downstream targets P70S6K and 4EBP1	(65)
	HCC	AMPK activation augments cisplatin-induced growth inhibition of HCC under *in vitro* and *in vivo* conditions	(66)
	Ovary	Under *in vitro* normo-glycemic conditions metformin inhibits cell proliferation and migration, and induces apoptosis *via* AMPK activation and decreased trimethylation of histone H3 lysine 27 (H3K27me3)	(67)
	AML	Induces G0/G1 phase arrest, elevates the expression of p-AMPK, p53, p21^CIP1^ and p27^KIP1^, and inhibits the expression of CDK4 and CyclinD1in SKM-1 cells. AMPK knockdown in SKM-1 cells reverses these effects.	(68)
	Pancreatic	Lowers the production of fibrogenic cytokines and prevents activation of pancreatic stellate cells. Metformin chemosensitizes gemcitabine to pancreatic cancer *in vivo.*	(69)
Phenformin	Breast	*In vivo*, biguanides inhibit local and metastatic growth of triple negative and HER2^+^breast cancer in immune-competent and immune-deficient orthotopic mice models. Biguanides inhibit local and metastatic breast cancer growth in a genetically engineered murine model of HER2^+^breast cancer	(70)
	Bladder	Inhibits cell migration and promotes apoptosis *via* activation of AMPK and inhibition of EGFR signaling. Phenformin exhibits synergism with gefitinib.	(71)
	Cholangiocarcinoma	Inhibits cell proliferation, migration, invasion, and angiogenesis by modulating AMPK-mTOR and HIF-1α-VEGF pathways.	(72)
	Lung	Phenformin functions as a radiosensitizer against non-small cell lung cancer cells.	(73)
	Acute Lymphoblastic Leukemia/Lymphoma	T-cell-specific loss of AMPK-α1 accelerates growth of T cell acute lymphoblastic leukemia/lymphoma. Phenformin delays the onset and growth of lymphomas, in the presence of AMPK-α1	(56)
AICAR	Glioblastoma	Inhibits the growth of EGFRvIII-expressing glioblastomas by retarding lipogenesis	(74)
	Retinoblastoma	AMPK activation negatively affects the growth and survival of retinoblastoma cells	(75)
	Thyroid	AMPK-activation impedes CXCL8 secretion and inhibits CXCL8-induced neoplastic cell migration	(76)
	Gastric	AICAR heightens 5-FU-induced apoptosis in gastric cancer cells	(77)
	Colorectal	AICAR chemosensitizes colorectal cancer cells to 5-Fluorouracil treatment	(78)
	Prostate	AICAR-mediated induction of apoptosis and prevention of migration and invasion is dependent on the AMPK/mTOR axis. AICAR radiosensitizes prostate cancer cells to radiotherapy. AICAR induces necrosis in prostate cancer cells in an AMPK-independent manner. AMPK activation induces apoptosis of DU-145 cells *via* generation of ROS and activation of c-Jun	(74, 79–81)
	Osteosarcoma	Induces mitochondrial apoptosis in an AMPK-dependent manner	(82)
Methotrexate	Breast, HCC	Methotrexate and AICAR exert synergistic anticancer effects against human breast cancer and hepatocellular carcinoma cells	(83)
	Breast	Co-treatment of AICAR and Methotrexate enhances mitochondrial oxidation and decrease the rate of glycolysis thereby blocking G1/S and the G2/M transition in the cell cycle.	(84)
Salicylate	Lung, Prostate	Salicylate and metformin synergistically reduce the survival rate of prostate and lung cancer cells *ex vivo via* inhibition of *de novo* lipogenesis	(85)
	Prostate	Metformin-salicylate treatment enhances sensitivity of prostate cancer cells to radiotherapy	(86)
	Breast	Salicylate-mediated AMPK activation down-regulates HAS2 and inhibits the metastatic potential of breast cancer cells	(87)
Canagliflozin	Glioblastoma	Inhibits growth and proliferation of glioblastoma cells by activating AMPK	(88)
	Liver	Inhibits glycolytic metabolism, induces G2/M arrest and apoptosis. Canagliflozin inhibits growth of subcutaneous xenografts and prevents intratumor vascularization independent of the glycemic status in BALB/c nude mice	(89)
	Colon	Decreases the number of BrdU positive cells and suppresses 4EBP1 and mTOR activity in an AMPK-dependent manner.	(49)
	Lung	Chemosensitizes NSCLC to both radiotherapy as well as chemotherapy	(86)

## Role of AMPK in cancer metabolism

Cancer cells require an unquenched and persistent supply of energy and nutrients to aid their survival and rapid rates of proliferation. There is an ever-growing need for the uninterrupted functioning of anabolic pathways for promoting cell growth in cancer cells. This includes pathways involved in the synthesis of glucose, fatty acids, phospholipids, protein, and ribosomal RNA. Cancer cells undergo metabolic reprogramming in order to meet the increased metabolic needs and ensure continuous growth and proliferation (90, 91). One of the characteristic traits of cancer cells is aerobic glycolysis, also referred to as the “Warburg effect” wherein, cancer cells rely on cytosolic glycolysis over mitochondrial oxidative phosphorylation irrespective of oxygen supply (92). This selective preference for glycolysis despite its low ATP productivity is for procuring intermediates for anabolic processes, including biosynthesis of glycogen, amino acids, nucleic acids, and lipids as well as for ensuring the increased stability of the mitochondrial membrane in the cancer cells (93).In addition to the changes in the glycolytic phenotype, tumor cells also manifest mitochondrial energy reprogramming to achieve metabolic plasticity. Recent studies attribute tumor aggressiveness and chemoresistance to the hybrid glycolysis/OXPHOS (oxidative phosphorylation) phenotype of cancer cells. Aberration in tumor glucose metabolism and elevated rates of glycolysis contribute to intrinsic/acquired resistance to routinely used anticancer drugs. Thus, the regulation of tumor metabolism would be a promising therapeutic strategy irrespective of the cancer type (94). Activation of AMPK counteracts tumor progression through negative regulation of the Warburg effect of tumor cells (95).AMPK plays a crucial role in regulating carbohydrate, lipid and protein metabolic pathways (96). AMPK quintessentially halts all of these pathways, thereby depriving the cancer cells of energy and nutrients (97). Hence, AMPK functions as a metabolic tumor suppressor by modulating energy levels, enforcing metabolic checkpoints, and inhibiting cell growth (98).

### Glucose metabolism

AMPK influences glucose transporter (GLUT)-mediated trafficking of glucose across the plasma membrane. In addition, it promotes cellular glucose uptake by enhancing the mRNA expression of the genes encoding GLUT4 and hexokinase 2 and by mediating the translocation of GLUT4-containing intracellular vesicles across the plasma membrane. Previous studies have reported the role of AMPK in GLUT1/4 vesicle trafficking *via* the activation of thioredoxin-interacting protein (TXNIP) and Tre-2/Bub2/Cdc16 (TBC) domain family member 1 (TBC1D1) respectively (99). Once glucose is internalized, it is converted to glucose-6-phosphate by the action of hexokinases. Glucose-6-phosphate is further channelized to glycolysis, glycogen synthesis and pentose phosphate pathway. Of all the glucose metabolism pathways, AMPK plays an indispensable role in directly inhibiting aerobic glycolysis and glycogen synthesis (100). AMPK stimulates glycolytic flux through direct phosphorylation of PFKFB2 and PFKFB3 isoforms of the enzyme 6-phosphofructo-2-kinase/fructose-2,6- bisphosphatase (101). AMPK phosphorylation also culminates in the inhibition of gluconeogenesis-related enzymes such as glucose-6-phosphatase(G6Pase) and phosphoenol pyruvate carboxykinase (PEPCK) (102). The activation of AMPK impedes glycogen synthesis and activates the rate of glycogen breakdown by promoting the phosphorylation of glycogen phosphorylase (GP).Notably, AMPK activation also enhances insulin sensitivity, inhibits hepatic glucose production in the liver, stimulates glucose uptake in the skeletal muscles, and weakens proinflammatory changes (103).

### Lipid metabolism

The conversion of acetyl-CoA to malonyl-CoA is the rate-limiting step in the *de novo* synthesis of fatty acids. AMPK was initially identified as a kinase that phosphorylates and inhibits acetyl CoA carboxylase (ACC), the enzyme involved in the conversion of acetyl-CoA to malonyl-CoA (104). AMPK activation leads to the inhibitory phosphorylation of sterol regulatory element-binding protein 1c (SREBP1c). SREBP1cis responsible for enhancing the production of lipogenic enzymes, such as, acetyl-CoA carboxylase 1 (ACC1) and fatty acid (FA) synthase. Further, AMPK negatively regulates the *de novo* synthesis of cholesterol and triglycerides, and promotes β-oxidation of fatty acids. Additionally, AMPK negatively regulates the first committed step in triglyceride (TG) synthesis that is catalyzed by the enzyme, glycerol-3-phosphate acyltransferase (GPAT). AMPK- mediated inhibition of cholesterol synthesis is induced by phosphorylation of the rate-limiting enzyme 3-hydroxy-3-methylglutaryl-coenzyme A (HMG-CoA) reductase (HMGCR). Both HMGCR and hormone-sensitive lipase (HSL) enzymes, which are involved in the synthesis of cholesterol and prevention of lipolysis, respectively, are inhibited as a consequence of AMPK activation (105). The role of AMPK in accelerating lipid catabolism is equally important. AMPK facilitates transportation of fatty acids into the mitochondria, where they are subjected to β-oxidation by the enzyme carnitine palmitoyltransferase-1 (CPT-1) (106, 107). Activation of AMPK accelerates the oxidation of fatty acids by inhibiting malonyl Co A and heightens the activity of CPT-1 enzyme (108).

### Protein metabolism

AMPK exercises its inhibitory effects on protein synthesis mainly through the inhibition of mTOR (12). mTOR is a serine/threonine protein kinase that regulates two distinct down-stream catalytic protein complexes, namely, mTOR complex 1 (mTORC1) and mTOR complex 2 (mTORC2) (109). mTORC1 plays an important role in the growth of cancer cells and in cell division. The major down-stream targets of mTOR that are involved in the translation machinery include p70S6 kinase (p70S6K) and 4E-binding protein (4E-BP). It is well known that eIF4G is a binding site on eIF4E, which is essential for translation initiation (110). However, when 4E-BP is phosphorylated by mTOR, it can no longer bind eIF4E. Various stress factors inhibit mTORC1 activity, which results in the phosphorylation of 4E-BP and in turn, prevention of the translation initiation factor eIF4E (111). AMPK indirectly causes blockade of cap-dependent protein translation by inhibiting mTORC1 expression. This is achieved by phosphorylating tuberous sclerosis complex 2 (TSC2) and regulatory- associated protein of mTOR (RPTOR). Upon inhibition of mTORC1, 4EBP1 gets activated and a simultaneous decrease in the levels of p70S6K is observed. Consequently, the initiation of cap-dependent translation of proteins is halted. Besides, AMPK-mediated activation of eukaryotic elongation factor 2 kinase(eEF2K)also blocks protein synthesis (112). Furthermore, AMPK retards ribosomal RNA synthesis *via* the inhibitory phosphorylation of transcription initiation factor 1A (TIF-1A). The translational elongation is blocked by AMPK through the phosphorylation of eEF2K, which inhibits eEF2.

### Mitochondrial biogenesis

Mitochondrial biogenesis replaces fresh mitochondria once the damaged mitochondria are eliminated *via* autophagic degradation. The process of mitochondrial biogenesis is vital for energy production and cellular response under nutrient-limiting conditions. Interestingly, the substrates for mitochondrial metabolism are generated as part of the autophagic clearance program. Previous research suggests that AMPK regulates mitochondrial biogenesis by regulating peroxisome proliferator-activated receptor-γ coactivator 1-α (PGC1α) (23). It is one of the cofactors which facilitate transcription of mitochondrial genes. AMPK influences PGC1α either by phosphorylating it directly or deacetylating it in a Sirtuin (silent mating type information regulation 2 homolog) 1(SIRT1)-dependent manner. Besides, AMPK-dependent increase in NAD^+^/NADH ratio or high levels of nicotinamide phosphoribosyl transferase(NAMPT) expression also lead to an augmented expression of PGC1α (113). AMPK-mediated mitochondrial biogenesis also accelerates the rates of ATP production. This is achieved by increasing the replication of mitochondrial DNA as well as the expression of many nuclear-encoded mitochondrial proteins *via* the SIRT 1/PGC-1α axis (114). Shen et al., have reported that the steroid hormone, ouabain, induces the simultaneous activation of AMPK and Src pathways in A549 and MCF7 cells and inhibits the mitochondrial OXPHOS in the cancer cells (115).

**Figure 3:**
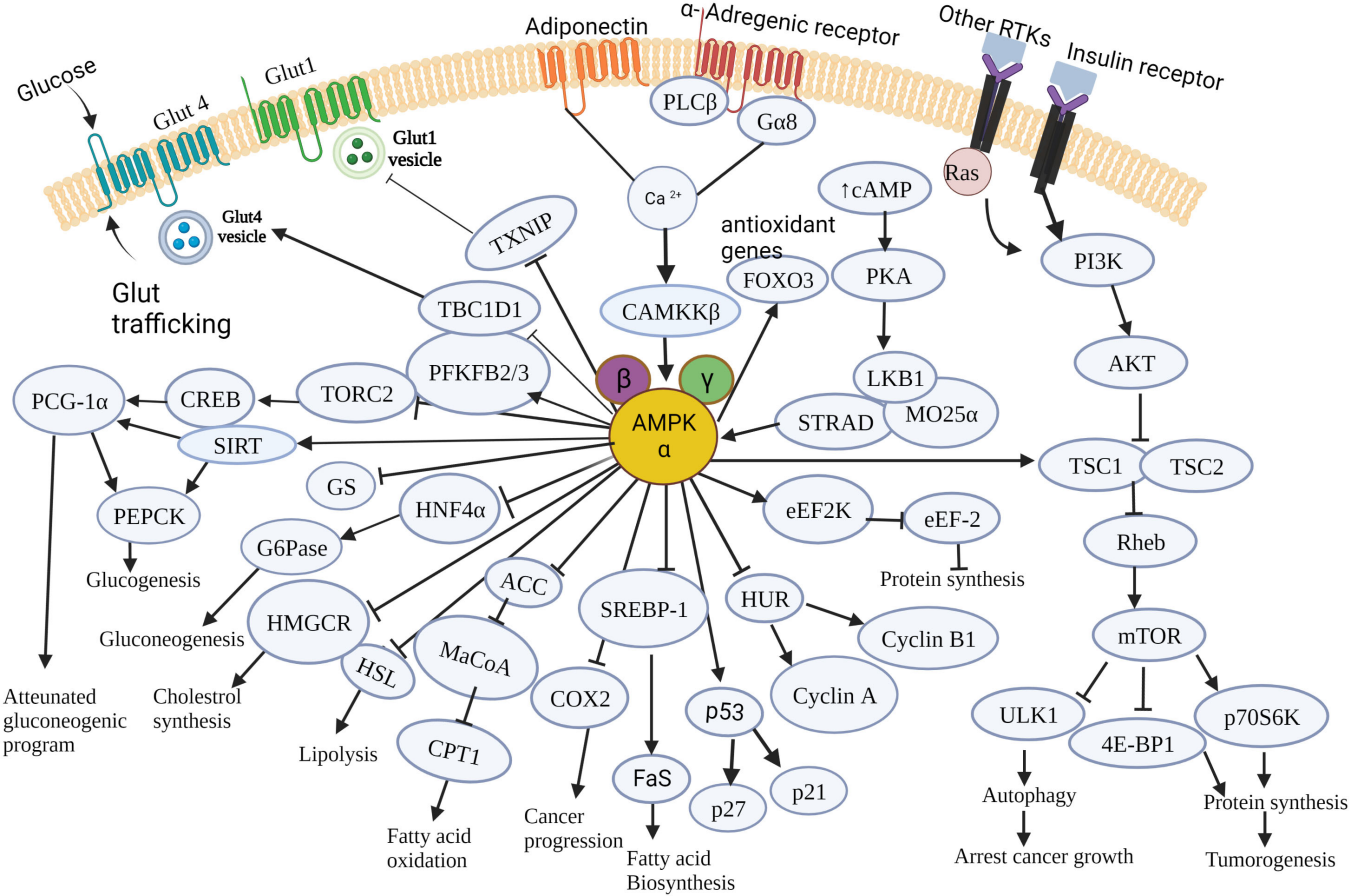
Schematic illustration of the major molecular targets of AMPK and the down-stream signaling events orchestrated by AMPK in cancer cell metabolism. AMPK controls various metabolic pathways that are critical to tumorigenesis and cancer progression. It regulates major cellular events governing cancer cell growth, survival and proliferation and pathways involved in glucose, lipid and protein metabolism. AMPK, AMP-activated protein kinase; mTOR, mammalian target of rapamycin; Glut4, Glucose transporter type 4; Glut1, Glucose transporter type1; RTK, Receptor tyrosine kinase; PLC-β, Phospholipase C- β; G6Pase, Glucose -6-phosphatase; HMGCR, Hydroxymethyl glutaryl-CoA reductase; HSL, Hormone sensitive lipase; cAMP, Cyclic adenosine 3’,5’-monophosphate; PI3K, Phosphoinositide 3-kinase; Akt, A protein-serine/threonine kinase; CAMKKβ, Ca^2+^/Calmodulin-dependent protein kinase kinase 2; PGC1α, PPARG coactivator 1 α; HNF4α, hepatocyte nuclear factor 4 alpha; ACC, acetyl-CoA carboxylase; MaCoA, malonyl CoA; CPT1, Carnitine palmitoyl transferase 1; TSC1, Tuberous sclerosis complex 1; TSC2, tuberous sclerosis complex 2; Rheb – Ras homolog enriched in brain; SREBP1, Sterol regulatory element – binding protein 1; COX2, Cyclooxygenase-2; FaS, Fatty acid synthase; ULK1, unc-51-like kinase 1;p70S6K,70-kDa ribosomal protein S6 kinase; 4EBP1, eIF4E-binding protein 1;HuR,Human antigen R;LKB1,Liver kinase B1; eEF2K,eukaryotic elongation factor 2 kinase; eEF2K, eukaryotic elongation factor 2; PFKFB2/3,6-phosphofructo-2-kinase/fructose -2,6 –biphosphatase 2/3; TBC1D1- tre-2/USP6,BUB2,cdc16 domain family member 1;TXNIP, Thioredoxin interacting protein; CREB, cAMP response element - binding protein; SIRT, Sirtuin; PEPCK, Phosphoenol pyruvate carboxykinase; TORC2, target of rapamycin complex 2; STRAD, STE20-related adaptor protein; MO25α, Mouse protein 25 α; FOXO3, Forkhead box family transcription factor 3; PKA, cAMP-activated protein kinase; GS, Glycogen synthase; Ras, rat sarcoma viral oncogene.

**Figure 3** summarizes the effects of AMPK on the modulation of cancer cell metabolism, which collectively contributes to cell death, cell growth-arrest, inhibition of tumorigenesis and tumor progression.

## The pro-tumorigenic functions of AMPK: Double-edged sword in cancer

Despite the accumulated observations supporting the tumor suppressing role of AMPK, there exist conflicting views on the pro-tumorigenic and pro-neoplastic features of AMPK. The tumor-suppressive function of AMPK might be overpowered by intracellular stress or oncogenic signals in malignant cancer cells (116).For instance, previous reports suggest that the activation of AMPK as a result of stress induced by glucose depletion or hypoxia enables tumor cells to become more resistant to metabolic stress (113, 117–119). Besides, AMPK-mediated autophagy confers a pro-survival advantage to cancer cells. Autophagy promotes cell growth and survival by providing metabolic substrates for biosynthesis, thus, fulfilling the metabolic demands of proliferating cancer cells. Besides, autophagy is also responsible for inducing chemoresistance in cancer cells (120, 121). There are also reports suggesting that although mTORC1inhibition prevents protein synthesis and cell proliferation, mTORC2 may activate the PI3K-Akt signaling pathway and promote tumor survival (106). A study by Laderoute et al, reports that AMPK promotes the growth of orthotopic tumors excised from breast cancer cells but does not affect the proliferation or survival of these cells *in vitro*. Experiments utilizing [^13^C] glucose tracers indicated that AMPK supports tumor glucose metabolism by combining the glycolytic and non-oxidative pentose phosphate cycles (122). Studies on astrocytoma murine models reveal that AMPK is an inducer of tumor cell proliferation. Furthermore, an elevated level of activated AMPK was found in human glioblastoma, in which inhibition of AMPK resulted in a significant decrease in the tumor cell growth rate (123). These results suggest that AMPK is not only involved in the regulation of ATP levels in cancer cells, but also in the regulation of cell replication. AMPK is required for increased mitochondrial biogenesis in response to glucose limitation. Studies by Chaube et al. suggest that under glucose-limiting conditions, cancer cells achieve metabolic homeostasis and adapt to metabolic stress *via* the activation of AMPK-p38-PGC-1*α* axis (124).Additional reports suggest that under conditions of nutrient starvation, AMPK may exhibit pro-tumorigenic effects and aid tumor survival, whereas in the presence of sufficient nutrients, AMPK exhibits tumor suppressing effects. The aforementioned data indicates that the pro- or anti-tumorigenic roles of AMPK are likely to be dependent on the levels of the nutrients in the TME (125). Notably, another independent study involving a pan-cancer analysis using multi-omics approach reveals that genetic as well as transcriptional aberrations in AMPK signaling elicits tissue-dependent pro- or anti-tumor impacts across major cancer types (126).

### Exploiting the pro-tumorigenic function of AMPK in anti-cancer therapy

Some of the recent studies throw light on the anti-cancer effects of pharmacological inhibitors of AMPK. Dorsomorphin or Compound C, the only known AMPK-specific inhibitor augmented the anti-cancer effects of Aspirin against HER-2-positive breast cancer in an AMPK-independent manner, by regulating lipid metabolism mediated by c-myc (127). Another study identified BAY-3827 as a novel AMPK inhibitor. The compound also inhibited ribosomal 6 kinase (RSK) family proteins.BAY-3827 displayed excellent anti-proliferative effects against androgen-dependent prostate cancer cell lines by blocking HMGCR, fatty acid synthase (FASN) and PFKFB2, all of which are strongly up-regulated by androgen treatment (128). Thus, it would be safe to say that AMPK is a potential molecular target for cancer therapy employing chemoprevention and chemosensitization approaches, irrespective of its individual pro- or anti-tumorigenic/neoplastic effects.

## Molecular crosstalk of AMPK drives anti-tumor immune response at the TME

The TME comprises of a heterogeneous collection of infiltrating and resident host immune cells, secreted factors, blood and lymph vessels, fibroblasts, endothelial cells, extracellular vesicles, and extracellular matrix. The stromal components together with the tumor cells are collectively referred to as the tumor microenvironment and its composition varies across different types of tumors (129). The TME facilitates cell survival and proliferation and promotes angiogenesis, local invasion, and metastasis of cancer (130–132). It includes cellular components of adaptive immunity, namely, T lymphocytes, dendritic cells (DC), and B lymphocytes, as well as those of innate immunity, including, macrophages, polymorphonuclear leukocytes, and natural killer (NK) cells, all of which exhibit context-dependent pro- or anti-tumorigenic functions (133). The metabolic changes occurring at the TME dictates the phenotypic and functional reprogramming of these immune cells. Immunotherapeutic drugs function by targeting specific components of the TME and shifting them from a pro-tumorigenic to an anti-tumorigenic phenotype (134). Owing to its diverse functions in regulating fundamental cellular activities, AMPK is instrumental in tumor metabolic transformation and controlling the metabolic plasticity of various immune cell types within the TME, which in turn potentiates an anti-tumor immune response (132).

### T cells

T cell activation in the TME is governed by various metabolic pathways such as, aerobic glycolysis, amino acid metabolism, glutaminolysis, and *de novo* fatty acid synthesis. The LKB1-AMPK signaling pathway stimulates catabolic pathways and ATP generation which in turn, facilitate metabolic reprogramming in T cells. AMPK enhances glutaminolysis and mitochondrial OXPHOS which aids in T cell survival. AMPK activation promotes fatty acid oxidation (FAO). The AMPK-mTOR axis is responsible for the generation of memory T cells (T_mem_)and adaptation of effector T cells (T_eff_) to nutritional stress (135). The inhibition of glycolysis and concomitant activation of FAO and oxidative phosphorylation directs regulatory T cells (T_regs_) to undergo metabolic reprogramming which culminates in T_regs_-mediated immunosuppression and tumor progression. On the contrary, cytotoxic CD8^+^ T cells play a crucial role in tumor immunosurveillance and elicit anti-tumor immune response by secreting interferon (IFN)-γ and granzyme B (GZB) (136).Rao et al., have demonstrated that AMPK regulates protein phosphatase activity, which controls survival and function of CD8^+^ T cells, thereby enhancing their role in tumor immunosurveillance (95). Besides, AMPK is indispensable for the sustained long-term proliferation of T cells and the survival of effector/memory T cell populations (137). In particular, AMPK promotes the accumulation of effector/memory T cells in competitive homeostatic proliferation settings (138). AMPK is found to be activated in CD8^+^ T cells during primary immune response. It also helps effector T cells (T_eff_) in their metabolic adaptation in response to reduced glucose availability. T_eff_ cells actively engage in glutamine-dependent oxidative phosphorylation (OXPHOS) to maintain ATP concentrations and cell viability under low glucose conditions. Further, AMPKα1-deficient T_eff_ cells display reduced mitochondrial bioenergetics and cellular ATP in response to glucose limitation (139). Notably, cytotoxic T cells (CD8^+^ T cells) detect abnormal tumor antigens expressed on cancer cells and target them for destruction. AMPKα-1-mediated inhibition of mTORC1 activity in cytotoxic T lymphocytes (CTLs) is required for CD8^+^ T-cell memory (140). Previous studies have revealed that inactivation of both AMPK*α*1 and *α*2 coupled with Kirsten rat sarcoma virus (KRAS) activation promotes tumorigenesis and decreases the infiltration of CD8^+^/CD4^+^ T cells (141). Braverman et al., have reported that increasing AMPK activity orchestrates oxidative metabolism, proliferation, and *in vitro* recovery of human CD4^+^ T cells. They also suggest AMPK as a potential candidate for improving the yield of more functional T cells for CAR-T cell therapy (142). The metabolic fitness of T cells is pivotal for antitumor immunity. A recent report suggests that AMPK is essential for the sustained long-term proliferation of T cells and the survival of effector/memory T cell sub-populations (143). However, under conditions of restricted nutritional supply, T cells acquire an exhausted phenotype inside the immune-suppressive tumor milieu. T cell exhaustion culminates in the loss of effector activities and changes in T cell signaling and is characterized by reduced rates of glycolysis and OXPHOS and the onset of mitochondrial dysfunction (135).

### Macrophages

Tumor-associated macrophages (TAMs) are present in abundance across various types of malignant tumors and promote tumor angiogenesis, extravasation and metastasis (144).Macrophages are responsible for driving anti-tumor immunity *via* phagocytosis and antigen presentation. Macrophages exist in two distinct phenotypes namely, the ‘classically’ activated, tumoricidal phenotype, M1φ, and the ‘alternatively’ activated pro-tumorigenic phenotype, M2φ (145).Some documented results indicate that AMPK inhibition leads to an up-surge in LPS-induced macrophage inflammatory function (141). Previous reports also state that AMPK prevents the polarization of monocyte-derived macrophages towards the M2φ subtype (146).Chiang et al., reported that metformin-mediated AMPK activation suppresses the skewing of macrophage towards M2φ subtype in breast cancer cells (147). The metabolic control of macrophage polarization relies partially upon glycolysis. A shift towards increased glycolytic rates leads to the activation of M1φ and is governed by the activation of mTOR through the Akt-HIF-1α pathway. Conversely, M2φ selectively utilize FAO, instead of aerobic glycolysis, to meet the energy requirements of OXPHOS. While HIF-1α and NF-κB favour the M1φ, PGC1β, peroxisome proliferator-activated receptors (PPAR) and STAT6 skew the balance towards M2φ. AMPK influences M1/M2φ polar mitochondrial biogenesis of macrophages by deacetylating proteins such as, SIRT1 with NAD^+^, and suppressing HIF-1α and NF-κB (1).

### Cytokines and chemokines

Previous reports also suggest that AMPK is involved in the molecular crosstalk between macrophages and cytokines. For instance, studies on the effect of macrophage stimulation using anti-inflammatory cytokines causes rapid phosphorylation of AMPK as opposed to the inactivation of AMPK upon the stimulation using pro-inflammatory LPS stimulus. AMPK directs signaling pathways in macrophages in a manner that suppresses pro-inflammatory responses and promotes macrophage polarization to an anti-inflammatory functional phenotype (146). AMPKα silencing elevated the mRNA expression levels of LPS-induced TNF-α, IL-6, and cyclooxygenase-2. Similarly, transfection of dominant negative AMPKα1 gene increased TNF-α and IL-6 expression levels and down-regulated IL-10 expression in macrophages, upon LPS stimulation (147).Another recent study revealed that AMPK inactivation potentiates the development of LPS-induced inflammatory injury (148). In the TME, AMPK has been reported to suppress the secretion of various pro-inflammatory cytokines such as IL-1β, IL-6, and TNF-α, interleukins-1/2/8, MCP-1, IFN-γ and chemokines such as RANTES, CCL 1/2/5/10/11 (148).Previous studies indicate that stimulation of wild type macrophages using anti-inflammatory cytokine,IL-10 results in rapid activation of AMPK, phosphoinositide 3-kinase(PI3K) and mTORC1 (149).AMPK is known to inhibit the functions of pro-inflammatory molecules such as IL-1 β. Previous reports suggest that AMPK activation inhibited IL-1-stimulated CXCL10 secretion, *via* the down-regulation of the MKK4/JNK and IKK/IκBα/NF-κB signaling axis (150).

### Myeloid-derived suppressor cells

Myeloid-derived suppressor cells (MDSC) are an immunosuppressive class of immune cells that are pathologically activated in various tumor types. MDSCs promote tumor progression and enhance tumor cell survival, angiogenesis, invasion, metastasis and production of immunosuppressive cytokines such as IL-10 and TGF-β. Recent studies indicate that splenic MDSCs impede T cell response in a ROS-dependent manner, whereas, tumor-infiltrating MDSCs inhibit anti-CD3/28-stimulated response through nitric oxide (NO) production and secretion of Arginase1 (Arg1). MDSCs mediate the addition of nitrate groups to chemokines thereby, blocking CD8^+^ T cells from entering into the tumor site (151). AMPK block the expansion and activation of MDSCs. It curtails the function of MDSCs *via* inhibition of JAK-STAT, NF-κB, C/EBPβ, CHOP, and HIF-1α pathways that are crucial for the development and migration of MDSCs (152–154).

### Immune checkpoint molecules

**Figure 4:**
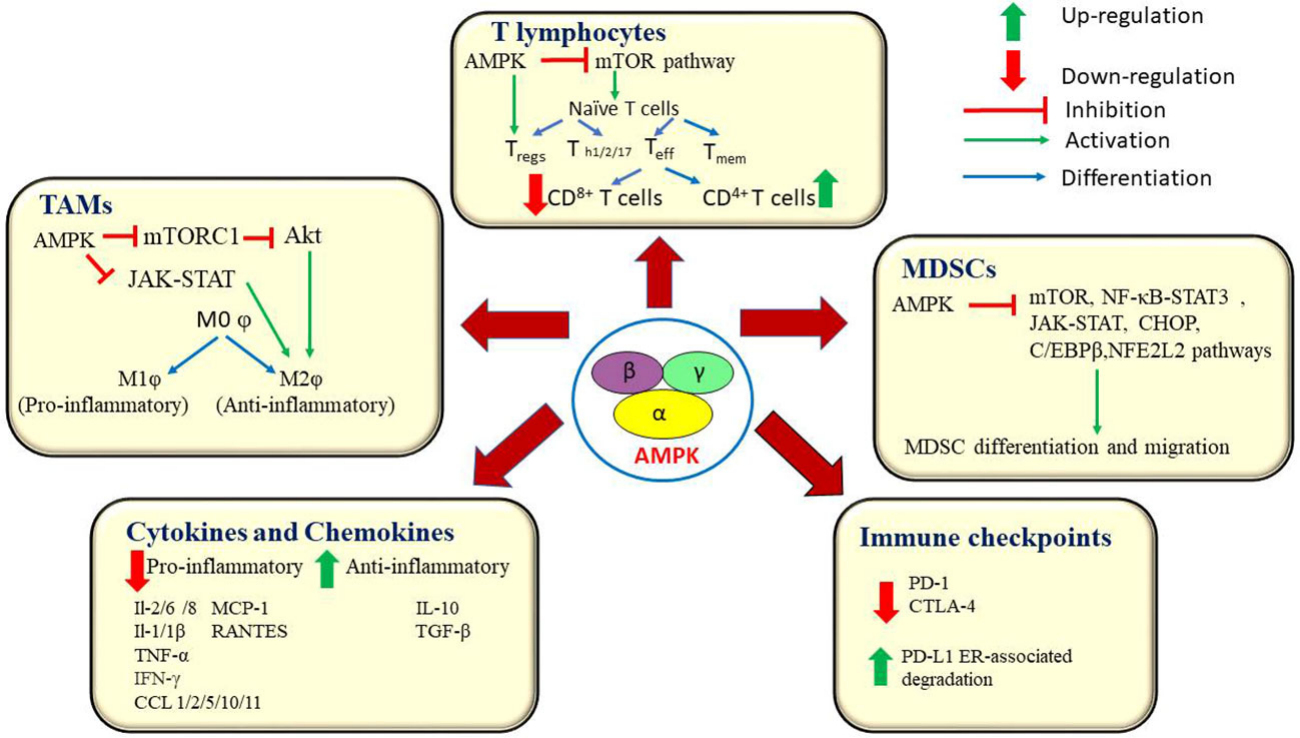
AMPK orchestrates an anti-tumor immune response by interacting with key players at the tumor microenvironment. AMPK influences the functions of T lymphocytes, macrophages and myeloid-derived suppressor cells. AMPK down-regulates the production of pro-inflammatory cytokines and chemokines and blocks the immune checkpoint molecules, PD-1 and CTLA-4. AMPK, AMP-activated protein kinase; mTOR, mammalian target of rapamycin; Akt, A protein-serine/threonine kinase; mTORC1, mammalian target of rapamycin complex 1; T_eff_, Effector T cells; T_mem_, Memory T cells; T_reg_, Regulatory T cells; T_h1/2/17_, Helper T cells1/2/17; PD-1, programmed cell death 1; IL-2/6/8/1/1β, Interleukin 2/6/8/1/1β; TNF α, Tumor necrosis factor α; IFN α, interferon gamma; PDL1, Programmed cell death ligand 1; CTLA-4, Cytotoxic T-lymphocyte – associated antigen 4; MCP-1, Monocyte chemoattractant protein -1; RANTES, Regulated upon activation, normal T cell Expressed, and Secreted; TGF-β, Transforming growth factor; MDSCs, Myeloid – derived suppressor cells; NF-κB, Nuclear factor kappa B; JAK/STAT, Janus kinase/signal transducers and activators of transcription; C/EBPR, CCAAT/enhancer – binding protein beta; CCL1/2/5/10/11, Chemokine (C-C motif) ligand 1/2/5/10/11; CHOP, C/EBP homologous protein; NFE2L2, Nuclear factor erythroid-derived 2-like 2; MOφ, non – activated macrophage; M1φ, pro-inflammatory macrophage; M2φ, anti-inflammatory macrophage.

The influence of AMPK on various immune checkpoints is yet another remarkable event in the alteration of the TME (116). Immune checkpoint molecules are co-stimulatory cell surface receptors that are expressed on the surface of several immune cells, which bind with their corresponding ligand molecules and in turn, prevent an immune attack against self-antigens. However, cancer cells use this feature of immune checkpoints to evade immune attacks. This confers a survival advantage to the cancer cells. Immunotherapy drugs aim at inhibiting these immune checkpoints rendering cancer cells susceptible to immune attacks (155).Blocking of the programmed cell death 1 (PD-1) receptor is currently being used as a first-line treatment option against lung cancer. A recent report indicates that GSK3β-mediated inhibition of glycogen synthase down-regulatesPD-1 expression levels on CD8^+^ T cells in B16F10, murine melanoma cells (136).Several reports also suggest the role of AMPK as an immune checkpoint inhibitor. AMPK activation causes phosphorylation of PD-L1 on Ser^283^ and disrupts its interaction with chemokine like factor (CKLF)-like MARVEL transmembrane domain containing 4 (CMTM4), which leads to the degradation of PD-1 ligand (PD-L1). AMPK also potentiates ER-associated degradation of PD-L1 (156, 157). Dai et al., have reported that, in syngeneic mouse tumor models, AMPK agonists enhance the efficacy of anti-CTLA-4 immunotherapy and improve the overall survival rate (156). Besides, reports also suggest the potential use of AMPK activators in combination with anti-VEGF/PD-1 agents as a dual-targeted therapy against ovarian cancer (116). A recent report by Pokhrel et al. states that AMPK drives anti-tumor immunity by blocking PD-1 expression *via* the HMGCR/p38 MAPK/GSK3β signaling pathway (136). They also report on the synergic antitumor effect of AMPK activators with anti-PD-1 antibodies, anti-CTLA-4 antibodies, or HMGCR inhibitors in murine tumor models (158). Another study has documented the AMPK-mediated blockade of PD-1 through a reduction of tumor hypoxia (159). Taken together, these findings highlight the central role of AMPK in the inhibition of major immune checkpoints. The current knowledgebase can be further expanded by evaluating the therapeutic efficacy of immunotherapy drugs in combination with AMPK inhibitors against multiple cancer types. **Figure 4** represents AMPK-mediated modulation of various components of the TME and the signalling pathways associated with it.

## Phytochemicals as natural activators of AMPK in cancer therapy

Even though the aforementioned chemical activators of AMPK show promising anti-cancer effects in the pre-clinical studies, there are drawbacks which prohibit their direct translation into the clinics. For instance, the synthetic biguanides, metformin and phenformin, were proposed to be used for the clinical management of type 2 diabetes in the 1950s. However, the clinical use of phenformin was withdrawn in the 1970s owing to a rare, but fatal condition induced by the drug known as ‘lactic acidosis’ (6). Mitochondrial impairment and ATP depletion leads to an acceleration of the glycolytic flux. Subsequently, there is an increased glucose uptake and excessive lactate generation. The excess lactate escapes into the circulation instead of undergoing further oxidation, giving rise to lactic acidosis (160). Although, metformin is safer than phenformin, it does induce lactic acidosis when used for long term. Incidences of such drug-induced toxicity and adverse side effects, and chemoresistance caused by conventional chemotherapeutic drugs limit the therapeutic efficacy of chemotherapy. The concept of chemoprevention and chemosensitization through dietary intervention has evolved to abate these detrimental effects. Over the past few decades, several studies have enumerated the immense therapeutic efficacy and pharmacological safety of various phytochemicals, highlighting them as suitable anti-cancer agents. Notably, many of these phytochemicals exert anti-cancer effects *via* the activation of AMPK, which results in increased apoptosis and inhibition of cell proliferation (161). Some of the phytochemicals that are well-known activators of AMPK include resveratrol, quercetin, berberine, ginsenoside, curcumin, epigallocatechin gallate(EGCG), theaflavin, hispidulin and galegine (6, 162). Recently, we have reported that Uttroside B, a novel saponin identified in our lab exhibits excellent anti-HCC effect *via* the up-regulation of AMPK and the concomitant down-regulation of mTOR (163). Taken together, substitution of phytochemical activators instead of chemical activators of AMPK would be a promising and pharmacologically safer strategy in treating different types of cancers. **Table 2** enlists some of the major phytochemicals which generate anti-cancer effects *via* AMPK activation.

**Table 2:** The implications of AMPK activation in various cancer types mediated by some of the major plant-derived anti-cancer agents.

Name of the phytochemical	Cancer Type	Effects of AMPK activation	References
Resveratrol	Colon	Induces apoptosis in chemoresistant HT29 cells	(164)
	Prostate	Sensitizes prostate cancer cells to ionizing radiation therapy	(165)
	Breast	Induces apoptosis in both ER-positive and ER-negative breast cancer cells in a SIRT1-dependent manner	(166)
	Ovary	Suppresses ovarian cancer growth and liver metastasis	(167)
Quercetin	Bladder	Induces apoptosis in both human and murine bladder cancer cells	(168)
	Colon	Induces apoptosis in HCT116 *via* Sestrin 2-AMPK-mTOR axis and increases intracellular ROS levels	(161)
Berberine	Melanoma	Decreases the metastatic potential of melanoma cells *via* inhibition of COX-2	(54)
	Colorectal	Inhibits mTOR and NF-κB activity and suppresses colon epithelial cell proliferation and tumorigenesis.	(169)
	Breast	Sensitizes drug-resistant breast cancer to doxorubicin chemotherapy	(57)
	Hepatocellular Carcinoma	Induces both apoptosis and autophagy in HepG2 cells.	(167)
	Colon	Inhibits NF-κB and MMP9 and in turn, suppresses colon cancer invasion	(170)
	Ovary	Induces cell death in CaOV3 cells in a p38-dependent manner	(57)
Uttroside B	Hepatocellular Carcinoma	Induces autophagy and apoptosis by modulating AMPK/mTOR axis, *in vitro* and *in vivo*	(163)
Emodin	Non-small-cell lung cancer	Increases expression of insulin-like growth factor binding protein 1	(171)
	Thyroid	Inhibits the proliferation of papillary thyroid carcinoma both *in vitro* and *in vivo*	(172)
EGCG	Breast	Suppresses breast cancer cell growth by inhibition of mTOR and activation of p21	(161)
	Colon	Inhibits COX-2 in HT-29 cells	(173)
Gingerol	Oral	Suppresses AKT/mTOR axis in YD10B and Ca9-22 cells	(174)
	Cervical	Inhibits PI3K/AKT and induces mTOR-dependent apoptosis in HeLa cells	(175)
	Osteosarcoma	Induces sub-G1 cell cycle arrest in osteosarcoma cells	(176)
Genistein	Hepatocellular Carcinoma	Down-regulates pro-inflammatory responses and attenuates liver damage	(177)
	Prostate	Induces antioxidant response in PC-3 cells	(39)
Ginsenoside	Breast	Inhibits cell proliferation and cell cycle progression in ER-positive breast cancer*, in vitro*.	(178)
Hispidulin	Hepatocellular Carcinoma	Inhibits tumor growth and lung metastasis, *in vivo*	(179)
	Renal, Prostate	Hispidulin synergizes with TRAIL and exhibits anti-cancer effects in both types of cancer, in an AMPK-dependent manner.	(180)
	Ovarian	Sensitizes ovarian cancer cells to TRAIL-induced apoptosis	(181)

## Limitations and future perspectives

Cancer cells being aberrant variants of normal cells, mimic normal cells to gain a survival advantage and escape cell death machinery and immune surveillance. This makes the eradication of cancer cells from the body a very challenging prospect. The unique metabolic features in the TME and cancer cells foster each other, resulting in sustained cell proliferation, tumor progression, and metastasis of cancer. Therefore, identifying molecules that act as focal points connecting cancer metabolism and anti-tumor immune response would be a promising strategy to combat cancer. In this scenario, AMPK acts as the nexus between cellular energy homeostasis, tumor bioenergetics, and anti-tumor immunity. Thus, AMPK would be a suitable candidate for targeted cancer therapy.

There are numerous pre-clinical evidences stating the dual nature of AMPK in cancer. However, the controversy over the positive and negative regulatory effects of AMPK in the context of cancer cannot be resolved without substantial clinical evidence (126). The research pertaining to the clinical outcomes of differential regulation of AMPK signaling in cancer is still in a nascent stage. Thus, it is quintessential to explore the causes and effects of the contradicting roles of AMPK in cancer progression using human tissue samples and PDX models of different types of cancer before drawing any conclusions. Future research aimed at elucidating the role of tumor type, tissue site and nutrient status of various cancer types in deciding the pro- or anti-tumorigenic functions of AMPK and the consequent clinical implication in patients is warranted.

Previous literature suggests that AMPK influences the differentiation and function of T cells and macrophages. A recent study revealed that AMPKα1 promotes mitochondrial homeostasis and persistence of B Cell memory although it hampers primary antibody responses (182). However, the literature on the role of AMPK in regulating the B cell metabolism and functions of B lymphocytes and humoral immunity is limited (1). Hence, a mechanistic evaluation of AMPK-mediated regulation of humoral immunity in various cancer types will facilitate in expanding the current knowledge.

AMPK-mediated disruption of cancer cell metabolism or alteration of the TME components to inhibit cancer progression would provide immense therapeutic benefits (183). These results would aid in the formulation of novel therapeutic regimen involving the activation or inactivation of AMPK, in a context-dependent manner, for mitigating cancer progression. Furthermore, substituting chemical activators/inhibitors of AMPK with their phytochemical functional equivalents may help circumvent the side-effects of chemo drugs without compromising their anti-cancer efficacy.

## Author contributions

RA conceptualized the work; CK, TR, SS, KK, and NA collected, analyzed, and interpreted the relevant literature and wrote the manuscript; CK prepared the tables; CK and SS prepared the figures; NA, KK, NI, and RA critically reviewed the manuscript. All authors contributed to the article and approved the submitted version.
